# Older adults and technology: in telehealth, they may not be who you think they are

**DOI:** 10.1186/s12245-017-0162-7

**Published:** 2018-01-03

**Authors:** Peter Greenwald, Michael Ethan Stern, Sunday Clark, Rahul Sharma

**Affiliations:** 1 Department of Emergency Medicine, NewYork Presbyterian-Weill Cornell Medical Center, 525 E 68 St. M-126, New York, NY 10021 USA; 2 Department of Emergency Medicine, NewYork Presbyterian-Weill Cornell Medical Center, 525 E 68 St. M-112, New York, NY 10021 USA; 3 Department of Emergency Medicine, NewYork Presbyterian-Weill Cornell Medical Center, 525 E 68 St. M-114, New York, NY 10021 USA; 4 Department of Emergency Medicine, NewYork Presbyterian-Weill Cornell Medical Center, 525 E 68 St. M-130, New York, NY 10021 USA

## Abstract

When we established an emergency department-based telemedicine program, we assumed that many older patients would be skeptical of the new technology and choose not to participate. Our assumption was incorrect. Of the 1052 patients we evaluated in the first several months, 355 (33%) were 60, 2 were 99. Satisfaction and quality assessment scores among older patients were similar to those for younger patients. Many of these older patients demonstrated flexibility and interest in the novel use of technology. Our emergency department-based telemedicine program resulted in safe and satisfactory care and was readily accepted by our older patients.

## Correspondence/Findings

Mrs. Taylor was in the emergency department and was talking to a doctor on a video screen. The first minute or two of the conversation had been a little strange, but the odd feeling went away after only a few minutes.

The population of older adults is rapidly growing. Similar to other age groups, the way older adults use technology is changing. Historically, older adults have been considered slower to use new technology than their younger compatriots. A common misconception is that, as consumers, older adults have either no interest in the use of technology or cannot use technology [1–3]. Data released by the Pew Research Center in 2017 suggests that this may not be accurate for an increasingly large number of older individuals. The younger old have Internet and broadband usage rates that approach those of the general population, and once Internet adoption occurs for this group, it often becomes an integral part of their daily lives [4]. While younger adults use a greater breadth of technologies for multiple uses, there is evidence that a more granular analysis reveals age-related differences in both usage and frequency of use that is dependent on the technology domain, with the greatest use of Internet use for older adults falling in communication, news, and community information domains [5]. The way older adults use new communication media is becoming increasingly important as connectivity to information, resources, and society plays an ever more central role in our lives and the lives of our older adult population. This connectivity is particularly salient in the realm of healthcare, where the use of technology to improve the efficiency, cost, and outcomes of care is gaining rapid traction.

Mrs. Taylor, a healthy 69-year-old with a past medical history of high blood pressure, had arrived in the emergency department complaining of knee pain. Four months ago, she had moved from California back to Brooklyn in order to be closer to her brother who was now in a nursing home. She was staying with her sister. Before returning to New York, she had been struck by a car and injured her right knee. She had visited an emergency department (ED) after the accident, an X-ray had been done, and she had been told the knee was not broken.

In the 4 months since she had been back in New York, the knee had become more swollen. In recent weeks, she had started using a cane. Her concern over her brother’s health had been taking up most of her time, and she had not been able to see a doctor or apply for New York State Medicare. She was worried because she was putting more weight on her bad left knee to stay off the right. She had had meniscal repair on the left knee 4 years earlier. In addition, she had run out of her blood pressure medication 2 months ago. When her sister, who was not in the best health either, began coughing with a fever, Mrs. Taylor helped her come to the ED.

After her sister was triaged and receiving care, Mrs. Taylor decided to see if she could get something done about her knee. She registered, and at triage, a physician assistant asked her if she would be willing to see a doctor over the video screen. She was told that the visit might be faster for her if she was seen by video. Mrs. Taylor was eager to get back to where her sister was, so she decided that she would try it.

A central tenet of geriatric medicine is the concept of the heterogeneity of the elderly [6, 7]. This is usually defined by the health differences inherent in age sub-groups within the older population but also may be applied to relationships with technology among the old. Some of these differences may divide along age, education, and socioeconomic status. Younger, more highly educated and affluent seniors use technology more readily and across broader platforms than the older old, who as a group tend to be less affluent, less educated, and often have a significantly greater burden of chronic illness and disability. The younger old group may have a more positive attitude toward the rewards of technology versus the older cohort, who are less connected to the technological world, both literally and psychologically.

Mrs. Taylor had a brief exam with the PA who checked her blood pressure and heart rate and listened to her lungs and heart. The PA also examined her knee. She was then taken to a room where she was asked to sit in a chair in front of a small table. There was a TV in the corner, artwork on the wall, and it was quiet. There were no other patients.

The TV made a sound, and a man appeared on the screen. It was strange seeing the doctor on the television. The doctor told her he could tell she looked uncomfortable and that he had been told she had knee pain. He asked her to tell him about what had been going on. Mrs. Taylor started to explain about the accident, about the worsening pain and swelling, and about how it was slowly becoming harder to walk. She described how, recently, she could feel and sometimes hear popping as she bent her knee. As she continued to talk, the oddness of speaking to the television monitor became less noticeable to her. She commented on how she “felt like she was having a real conversation.” The doctor adjusted the camera for a close-up of her knee and asked her to move her knee in different ways. He then asked her about her blood pressure. Mrs. Taylor had been expecting this and told the doctor about her old medication and how she had not been taking it for the past few months.

The doctor explained that the number was too high but that what was more important than one particular reading was what the blood pressure was day after day. He explained that the number was high enough that it would be a very good idea that she restart her medication. He also discussed his assessment of her knee pain and that he doubted there was an injury to her bone but rather that it was likely a problem involving the cartilage, similar to what she had had with her other knee. He explained that an X-ray would not say much about injuries to parts other than the bone and that she would probably need an MRI and follow-up with a doctor who specialized in knees. She asked the doctor where he was, and he explained that he was about a block away and that he was seeing patients in the hospital where she was located and, at the same time, he was seeing patients at another hospital in the city and also seeing patients calling in from home on their computers. He said he liked seeing people like this because he thought he could talk to them more “one-on-one” with fewer interruptions. Mrs. Taylor told the doctor that she thought they were having a better conversation than they would if they were in the emergency department (which she realized they still were). The doctor then called someone to come into her room who talked to her about making an appointment with a primary care doctor to make a plan for treating her blood pressure and also with an orthopedic clinic where they would do further evaluation of her knee. The doctor then printed out some papers in the room about both high blood pressure and knee pain. He stressed the importance of the appointments that were being set up for her, and he asked her if she had any questions. Mrs. Taylor asked the doctor briefly about her brother in the nursing home; she was not happy with the specialist there. The doctor talked to her about how she might ask for a second opinion. As Mrs. Taylor finished the visit, she looked at her watch. It had been a little over 35 min since she left her sister in the other part of the ED.

Healthcare-related technology has expanded to include behavior monitoring technology, smart home applications, and telehealth interventions; however, research in the use of technology in the healthcare domain by older adults is still in its infancy. A 2011 study investigated technology type and frequency of use for a range of healthcare activities and found that older adults used automated telephone menu systems, medical-related Internet purchasing (e.g., medical supplies or medications), and telemedicine videoconferencing with healthcare providers more frequently than younger adults [4]. Another study showed that without an active, in-person communication component, technology-based interventions (providing automated electronic alerts and reminders for refill of prescriptions) alone are not effective in improving medication adherence rates or patient cardiovascular outcomes [8]. An increasing number of healthcare systems have already implemented telemedicine video communication as a tool for both expedited consult services (stroke, trauma, mental health screening, surgical second opinions) and post-discharge health maintenance to reduce hospital readmissions. There have been mixed results in the evaluation of telehealth programs for adults, including the elderly, with chronic medical conditions. In diabetic patients, a positive impact was shown for both glycemic control and health service usage [9]. In a study of patients with COPD, diabetes, and heart failure, the majority (70%) of whom were > 65 years old, an assessment of the effect of home-based telehealth interventions on the use of secondary care and mortality resulted in reduced emergency admission rates and lower mortality; however, separate analysis of the same data showed no effect on psychological outcomes or quality of life over a 12-month period [10, 11]. Research on telemedicine-enhanced acute care for older adults has focused on residents of senior living community facilities and has demonstrated that high-intensity telemedicine services for acute illnesses were completed successfully, considered acceptable by older patients, and provided definitive care without requiring ED or urgent care use [12]. Very little is known, however, about older patients who receive care via an ED-based telemedicine intervention.

When we established a program to see low acuity patients in the NewYork Presbyterian-Weill Cornell Emergency Department by telemedicine, we assumed the majority of older patients who presented to the ED and who fit our inclusion criteria would choose not to be seen by telemedicine and that the vast majority of our telemedicine patients would be younger adults who we assumed would be more comfortable with the technology.

Despite having an established geriatric emergency medicine (GEM) program at our hospital (including a GEM fellowship, a strong didactic GEM track for our EM residency, and inclusion of GEM faculty in our telehealth program), our thoughts regarding the way the program would be received by older adults was based on an ignorant, and even ageist, misconceptions. Of the 1052 patients we evaluated in the first several months the program was active, 355 were 60 or older (median age for patients older than 60 was 72) and 2 patients were 99 years old. Older patients were no more likely to return to the ED within 72 h than were younger patients, and they had very high levels of satisfaction with their visits (98th percentile as measured by Press Ganey). Our observations and our data have refuted our initial assumption that this program would be most appropriate for the young. Many of the older patients we have cared for have demonstrated flexibility and interest in the novel use of technology. “It is always good to try something new” is a quote we have heard over the video monitor from more than one patient in their 90s.

In an excellent editorial titled, “Why the Telemedicine Physical is Better Than You Think,” the authors, Hollander and Joshi, discuss the value of a telemedicine exam, reminding us that the two most powerful tools the practice and art of medicine have always had and still have are (1) the history of presenting illness (or trauma) and (2) our practitioner powers of keen observation [13]. The rest of the current typical medical work-up (blood work, radiology, specialty consults, etc.) is, at the very best, useful adjunct information and, at worst, a diverting crutch that has become far too easily accessed and upon which we, healthcare providers, have become too accustomed to rely. Perhaps, the older population has intuitively understood some of the paradoxical virtues and power of technology in the form of a telemedicine encounter, namely, that the essence of care is the basic connectivity with another human being, the telling of one’s narrative of what is ailing them, and the trust that the person across from them (on the monitor) is listening and observing carefully. Their wisdom in embracing telemedicine is actually an understanding and faith that we will return to and excel at “old-school” medicine. Technology meets “retro” in the best sense.
